# Implication of Melanocortin Receptor Genes in the Familial Comorbidity of Type 2 Diabetes and Depression

**DOI:** 10.3390/ijms23158350

**Published:** 2022-07-28

**Authors:** Mutaz Amin, Jurg Ott, Rongling Wu, Teodor T. Postolache, Claudia Gragnoli

**Affiliations:** 1 Institut National de la Santé et de la Recherche Médicale (INSERM), US14-Orphanet, 75014 Paris, France; mtz88@hotmail.co.uk; 2 Department of Biochemistry and Molecular Biology, Faculty of Medicine, Al-Neelain University, Khartoum 11121, Sudan; 3 Laboratory of Statistical Genetics, Rockefeller University, New York City, NY 10065, USA; ott@rockefeller.edu; 4 Department of Statistics and Department of Public Health Sciences, Penn State College of Medicine, Hershey, PA 17033, USA; rwu@phs.psu.edu; 5 Mood and Anxiety Program, Department of Psychiatry, University of Maryland School of Medicine, Baltimore, MD 21201, USA; tpostola@som.umaryland.edu; 6 Rocky Mountain Mental Illness Research Education and Clinical Center (MIRECC), Veterans Integrated Service Network (VISN) 19, Denver, CO 80246, USA; 7 Military and Veteran Microbiome: Consortium for Research and Education (MVM-CoRE), Denver, CO 80246, USA; 8 Mental Illness Research Education and Clinical Center (MIRECC), Veterans Integrated Service Network (VISN) 5, VA Capitol Health Care Network, Baltimore, MD 21090, USA; 9 Division of Endocrinology, Department of Medicine, Sidney Kimmel Medical College, Thomas Jefferson University, Philadelphia, PA 19107, USA; 10 Division of Endocrinology, Department of Medicine, Creighton University School of Medicine, Omaha, NE 68124, USA; 11 Molecular Biology Laboratory, Bios Biotech Multi-Diagnostic Health Center, 00197 Rome, Italy; 12 Department of Public Health Sciences, Penn State College of Medicine, Hershey, PA 17033, USA

**Keywords:** depression, MDD, type 2 diabetes (T2D), melanocortin receptor gene, *MC1R*, *MC2R*, *MC3R*, *MC4R*, *MC5R*, hypothalamic–pituitary–adrenal axis (HPA-axis), linkage, linkage disequilibrium, association, comorbidity

## Abstract

The melanocortin receptors are G-protein-coupled receptors, which are essential components of the hypothalamic–pituitary–adrenal axis, and they mediate the actions of melanocortins (melanocyte-stimulating hormones: α-MSH, β-MSH, and γ-MSH) as well as the adrenocorticotropin hormone (ACTH) in skin pigmentation, adrenal steroidogenesis, and stress response. Three melanocortin receptor genes (*MC1R*, *MC2R*, and *MC5R*) contribute to the risk of major depressive disorder (MDD), and one melanocortin receptor gene (*MC4R*) contributes to the risk of type 2 diabetes (T2D). MDD increases T2D risk in drug-naïve patients; thus, MDD and T2D commonly coexist. The five melanocortin receptor genes might confer risk for both disorders. However, they have never been investigated jointly to evaluate their potential contributing roles in the MDD-T2D comorbidity, specifically within families. In 212 Italian families with T2D and MDD, we tested 11 single nucleotide polymorphisms (SNPs) in the *MC1R* gene, 9 SNPs in *MC2R*, 3 SNPs in *MC3R*, 4 SNPs in *MC4R*, and 2 SNPs in *MC5R*. The testing used 2-point parametric linkage and linkage disequilibrium (LD) (i.e., association) analysis with four models (dominant with complete penetrance (D1), dominant with incomplete penetrance (D2), recessive with complete penetrance (R1), and recessive with incomplete penetrance (R2)). We detected significant (*p* ≤ 0.05) linkage and/or LD (i.e., association) to/with MDD for one SNP in *MC2R* (rs111734014) and one SNP in *MC5R* (rs2236700), and to/with T2D for three SNPs in *MC1R* (rs1805007 and rs201192930, and rs2228479), one SNP in *MC2R* (rs104894660), two SNPs in *MC3R* (rs3746619 and rs3827103), and one SNP in *MC4R* genes (Chr18-60372302). The linkage/LD/association was significant across different linkage patterns and different modes of inheritance. All reported variants are novel in MDD and T2D. This is the first study to report risk variants in *MC1R*, *MC2R*, and *MC3R* genes in T2D. *MC2R* and *MC5R* genes are replicated in MDD, with one novel variant each. Within our dataset, only the *MC2R* gene appears to confer risk for both MDD and T2D, albeit with different risk variants. To further clarity the role of the melanocortin receptor genes in MDD-T2D, these findings should be sought among other ethnicities as well.

## 1. Introduction

The melanocortin receptors are G-protein-coupled receptors mediating the actions of melanocortins (melanocyte-stimulating hormones: α-MSH, β-MSH, and γ-MSH) and the adrenocorticotropin hormone (ACTH) in skin pigmentation, adrenal steroidogenesis, and stress response [1]. Melanocyte-stimulating hormones (α-MSH, β-MSH, and γ-MSH) and ACTH are synthesized in peptidergic neurons in the arcuate nuclei of the hypothalamus and the pituitary gland, respectively, from the same precursor: pro-opiomelanocortin (POMC) via post-translational modifications [2,3]. This central melanocortin system is part of the hypothalamic–pituitary–adrenal (HPA) axis, which is involved in stress responses [4] and metabolic regulation [5] and is expressed in both central (e.g., brain) and peripheral tissues (e.g., skin) [6]. There are five known melanocortin receptors in humans (*MC1R*-*MC5R*) [7]. *MC1R* is predominantly expressed in skin melanocytes, adrenal glands, kidneys, and immune cells [8,9]. *MC2R* is mainly expressed in the adrenal cortex [10]. *MC3R*, *MC4R,* and *MC5R* are expressed in the brain and other tissues (e.g., *MC3R* in macrophages [11]); *MC5R* is also present in adipose tissue, kidneys, and skeletal muscles [7,12].

The melanocortin receptors are encoded by five different genes (*MC1R*-*MC5R*) that exert different physiological functions in both humans and domestic animals [7]. *MC1R* is best known for regulating skin and coat pigmentation, and *MC2R* is the main receptor for ACTH [13]. Mutations in the *MC2R* gene can cause familial glucocorticoid deficiency [14]. *MC3R* and *MC4R* play important roles in energy and lipid metabolism [15]. *MC4R* dysfunction causes obesity in both humans [16] and knockout mice [17]. The role of *MC3R* in energy homeostasis is less clear, and *MC3R*-knockout mice have normal weight and normal or low appetite [18]. While the *MC5R* function is the least understood, the evidence so far suggests its role in energy metabolism, inflammatory responses, and exocrine functions [19].

T2D and MDD are two prevalent chronic complex diseases associated with significant worldwide morbidity and mortality [19]. They cumulatively affect 14% of adult populations [20,21], and their etiologies can be attributed to interactions between environmental and genetic risk factors [22,23,24]. Genetic overlap exists between MDD and T2D and can be linked to at least a few genes [25,26].

Melanocortin receptors mediate the action of the hypothalamic–pituitary–adrenal (HPA) axis in response to superimposed stresses and cortisol feedback, which have been linked to depression (MDD) [27] and type 2 diabetes (T2D) [28]. In humans, polymorphisms in the melanocortin receptor genes have been previously reported in patients with major depressive disorder (MDD) (*MC1R* [29], *MC2R* [30], and *MC5R* [31]), emotional eating and food craving (*MC4R* [32]), obesity (*MC1R* [33], *MC3R* [34], *MC4R* [12], and *MC5R* [12] via linkage studies in Quebec families [12]), and T2D (*MC4R*) [35], but never in the MDD-T2D comorbidity. In this study, we evaluate the contribution of variants in the melanocortin receptor genes to the familial comorbidity of T2D and MDD.

## 2. Results and Discussion

### Linkage, LD/Association Analysis, and LD among SNPs

We detected significant (*p* ≤ 0.05) linkage to and/or LD (i.e., association) with MDD for one SNP in *MC2R* and one SNP in *MC5R* and to/with T2D for three SNPs in *MC1R*, one SNP in *MC2R*, two SNPs in *MC3R*, and one SNP in *MC4R*. Table 1 shows information on the significant parametric models and chromosome and base pair location, Ref/Alt alleles and risk alleles, gene sites, and functional consequences of the specific risk variants. Moreover, Table 1 reports if the risk variant is independent or within a LD block, and whether it has been previously published in MDD or T2D.

**Figure 1:**
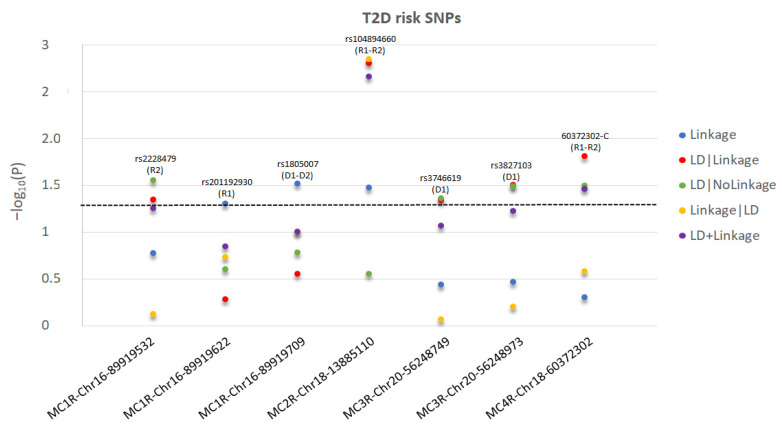
For each significant T2D-risk SNP in *MC2R*-*MC4R* genes, we present the −log_10_(P) as a function of each test statistic (Linkage, LD|Linkage, LD|NoLinkage, Linkage|LD, and LD+Linkage) and label the significant inheritance model: D1: dominant, complete penetrance; D2: dominant, incomplete penetrance; R1: recessive, complete penetrance; R2: recessive, incomplete penetrance. For *MC1R*-rs1805007, the most significant test statistics between D1 and D2 are presented. For *MC2R*-rs104894660, the most significant test statistics between R1 and R2 are presented. For *MC4R*-60372302-C, R2 test statistics are presented as more significant than R1. The level of statistical significance is marked by the dotted line.

**Figure 2:**
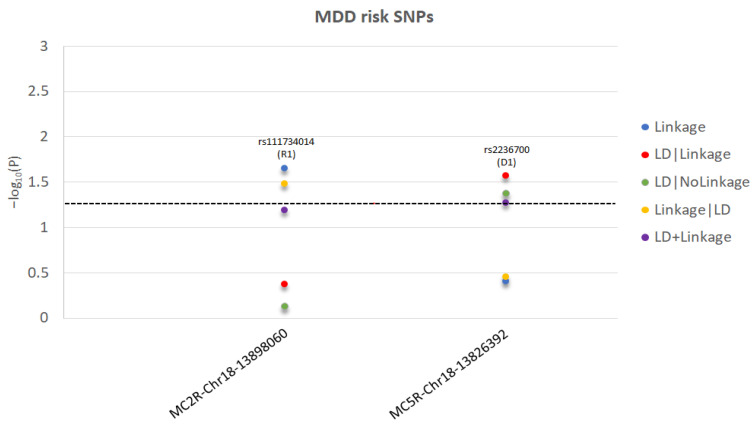
For each significant MDD-risk SNP in *MC2R* and *MC5R* genes, we present the −log10(P) as a function of each test statistic (Linkage, LD|Linkage, LD|NoLinkage, Linkage|LD, and LD+Linkage) and label the significant inheritance model: D1: dominant, complete penetrance; R1: recessive, complete penetrance. The level of statistical significance is marked by the dotted line.

The test statistics and specified significant models are reported in Figure 1 for T2D and Figure 2 for MDD. The *MC3R* risk variants rs3746619 and rs3827103 are within LD-block Set 01 (Table 1) and, thus, function as replicates of one another.

In this study, we reported nine variants in the melanocortin receptor genes that are significantly linked/in LD (or associated) to/with MDD and/or T2D in families with enriched T2D history. None of these variants have previously been reported in MDD or in T2D. This study pioneers the investigation of the five melanocortin receptor genes’ roles in familial MDD and T2D. Within our familial dataset, the *MC2R* and *MC5R* genes were significantly linked/in LD (or associated) to/with MDD, and four genes (*MC1R, MC2R, MC3R*, and *MC4R*) were significantly linked/in LD (or associated) to/with T2D. Of interest, the *MC2R* gene was significantly linked/in LD (or associated) to/with both MDD and T2D, thereby indicating the *MC2R* gene’s possible role in their comorbidity, despite being mediated by independent variants. To our knowledge, this study reveals a novel link of *MC1R*, *MC2R,* and *MC3R* genes to T2D.

The *MC1R* gene has been extensively studied in relation to skin and hair color [36]. Its role in MDD and obesity might be explained by ultraviolet light-induced mood changes [37]. However, *MC1R* has a role in mediating anti-inflammatory response. A study shows that both interferon and lipopolysaccharide (LPS) trigger *MC1R* expression in human neutrophils, and *MC1R* mediates the anti-inflammatory effects of alpha-MSH, likely contributing to neutrophil chemotaxis direct inhibition and anti-inflammatory activity [38]. Agonists of *MC1R* play a role in inflammatory response [39]. In mice, a melanocortin-like peptide blocks, as much as alpha-MSH and ACTH, cytokines’ release in response to LPS, rescuing the animals from lethal LPS doses, thereby showing that *MC1R* may play an anti-inflammatory role in protecting against LPS-generating gut microbes [40]. Thus, *MC1R* might play a systemic role in mediating inflammation derived from the gut–brain axis [41]. The role that *MC1R* plays in inflammation might contribute to T2D. In the present study, we found for the first time that the *MC1R* gene is related to T2D. In fact, we found *MC1R* rs1805007 significantly linked to T2D under the D1 and D2 models. It is known that *MC1R* rs1805007 regulates skin pigmentation [42] and is associated with red hair [43] and morbid obesity [33]; as in the present study, a prior study also failed to find that it confers MDD risk [29]. We also detected two additional *MC1R* variants. We found that the variant rs2228479 is linked/in LD (or associated) to/with T2D, specifically under the recessive incomplete penetrance model (R2); previously, it was associated with morbid obesity [33] and with antidepressant response in MDD patients [29]. Furthermore, we found that the rs201192930 G allele is linked/in LD (or associated) to/with T2D, specifically under the recessive, complete penetrance model (R1). Previously, the A allele was reported as contributing to melanoma [44].

*MC2R* binds to ACTH and mediates the release of cortisol from the adrenal glands [13]. Variants in the *MC2R* gene have been reported in Chinese patients with MDD [30]. In the Italian families under study, we found for the first time that *MC2R* variants confer risk for T2D. Namely, the study revealed that rs111734014 is significantly linked/in LD (or associated) to/with MDD under the recessive complete penetrance model (R1). Additionally, we detected that the *MC2R* rs104894660 G allele is significantly linked/in LD (or associated) to/with T2D, under the recessive complete (R1) and incomplete (R2) penetrance models. Of note, the *MC2R* rs104894660 A allele is reported in Clinvar and Uniprot as “pathogenic”, causing familial glucocorticoid deficiency via a recessive model of inheritance [45]. The *MC2R* rs104894660 G allele linkage/LD/association to/with T2D is, therefore, novel and might be explained by an increased *MC2R* affinity to ACTH, leading to higher cortisol secretion and subsequent insulin resistance, which together contribute to T2D. The potentially higher cortisol level can also explain the predisposition to stress-related MDD by its negative impact on mood, as it has been demonstrated in humans [46].

The *MC3R* gene is involved in obesity [47] and a marker near this gene has been reported having a role in insulin secretion [48]. Deficiency of *MC3R* in mice cause increased fat deposition and obesity, despite the decreased appetite [49]. Obesity may lead to T2D. In our study, we detected two *MC3R* closely linked variants, rs3746619 and rs3827103, contained within the same LD-block Set01, that are significantly linked/in LD (or associated) to/with T2D under the D1 model. As rs3746619 is located in the 5′UTR *MC3R* region and rs3827103 is a missense variant, their pathogenetic effect might be unrelated, as the first might affect gene transcription and the second may affect protein conformation. These two variants have been previously studied in obesity with inconsistent results. Both variants have been negatively associated with obesity in studies involving Caucasian [50], Chilean [51], and Thai [52] populations; rs3746619 has been positively associated with obesity in a study in a Singaporean [53] population; rs3827103 has been positively associated with obesity in a study in Caucasians [33] and with body fat percentage in a study in Malaysian adolescents [54] and African-Americans [55], implying a potential role in body fat composition in various ethnic groups [33]). These inconsistent results might be due to underlying allelic population differences specifically reported for these two variants [56], potential different LD blocks carrying the risk variant across populations, or differences in sample sizes and detecting power.

Variants in the *MC4R* gene—well-known as the human obesity gene [57]—have been reported in Chinese patients with T2D [35], and *MC4R* knockout mice are hyperphagic and obese [58] and have marked insulin resistance [59], all of which may contribute to T2D. We detected a novel variant, chr18-60372302-C, in linkage/LD/association to/with T2D under the recessive complete (R1) and incomplete (R2) penetrance models. The alternative allele T, not conferring risks within our families for T2D, causes a W16X stop variant that was previously reported in a mother and child with early-onset obesity, not confirmed in the other overweight/obese family members, and absent in the control subjects [60,61]. This T allele confers impaired *MC4R* expression and signaling, both in vitro and in vivo [62], while the stop signal is rescued in vitro by aminoglycoside-mediated read-throughs of stop codons [63].

The *MC5R* was the last of the melanocortin receptors to be cloned, and it is potentially implicated in energy metabolism and inflammatory responses [64]. Variants in the *MC5R* gene have been previously associated with MDD [31] and T2D in Finns [65]. We detected the *MC5R* rs2236700 SNP as significantly linked/in LD (or associated) to/with MDD under the dominant complete penetrance model. While a previous study reported no association of rs2236700 with bipolar disorder [66], interestingly, the rs2236700 T2D-risk G allele we detected confers susceptibility to schizophrenia when present with two variants of two other genes (tryptophan 2,3-dioxygenase [*TDO2*] and melanin-concentrating hormone receptor 2 [*MCHR2*]) [67]. Of note, the *TDO2* gene is activated by glucocorticoids [68] and is a candidate gene in other neuropsychiatric disorders (i.e., autism [69] and alcohol use disorder [70]), as it mediates immunosuppressive effects of kynurenine and its metabolites, loss of effective immune surveillance [71], and inflammation [72]. The pathogenic mechanism of *MC5R*-related MDD might be mediated by its role in inflammatory responses [64]; of note, *MC5R*-deficient mice display behavioral changes such as reduced aggression and more defensive behaviors [73]. Mutated *MC5R* in humans might cause similar behavioral and/or mood changes, but this remains to be confirmed.

As we and others have described [74,75], MDD, schizophrenia, bipolar disorder, and T2D share genetic comorbidity, but mental–metabolic comorbidity studies have begun in recent years [76,77]. Of equal interest, the globally recognized T2D risk gene *TCF7L2* [78] has been found via a linkage study to contribute to schizophrenia [79], further supporting the existence of comorbid genetic pathogenesis of metabolic–mental disorders.

Our study suggests that the melanocortin receptors risk variants, detected as contributing to familial risk for MDD and/or T2D, might be part of a more complex pathway implicated in the shared comorbidity of metabolic and mental disorders [76,77]. While it is hard to disentangle the genes’ direct roles in the phenotypes tested from the possible underlying biological effect(s), the genes reported appear implicated in the investigated phenotypes, but only *MC2R* shows pleiotropic effects within our familial dataset. This might be explained by the mediating effect of the HPA-axis on the *MC2R* of the adrenal, triggering cortisol secretion. Hypercortisolism is implicated in MDD [27] and T2D [28], and as we previously hypothesized, it is most likely implicated in the MDD-T2D comorbidity [25]. However, we want to note the significance and intrinsic limitation of the present study. While variants in linkage with a disorder cosegregate with the disease, they are not necessarily associated with it; on the other hand, variants in LD with a disease are both in linkage and associated with it; thus, they cosegregate as well as associate with the disease under study across various families. Despite this, only in vitro or in vivo studies can prove the functional effects of the variants on the gene expression, translation, or downstream function. Thus, we cannot prove that the detected risk variants are indeed causative variants; they might be in LD with an unknown, yet-to-be identified pathogenic variant.

## 3. Materials and Methods

Our aim was to investigate the potential role of the *MC1R*, *MC2R*, *MC3R*, *MC4R*, and *MC5R* genes in the pathogenesis of T2D, MDD, and their comorbidity.

We studied previously recruited Italian families with T2D, and the dataset was deidentified and coded. The study was approved by the Jefferson Ethical Committee. The 212 families studied descended from at least three generations of Italians originating from the Italian peninsula. Families with identical twins and siblings with uncertain paternity were excluded. The families had an enriched history of T2D [80,81] and were phenotyped for the presence or absence of MDD using DSM-IV diagnostic criteria [82].

In the family subjects, we amplified 11 single nucleotide polymorphisms (SNPs) in *MC1R*, 9 SNPs in *MC2R*, 3 SNPs in *MC3R*, 4 SNPs in *MC4R*, and 2 SNPs in *MC5R* using microarrays. We performed genotyping and Mendelian error exclusion by PLINK [83]. Using Pseudomarker, we analyzed the total 29 SNPs for 2-point parametric linkage and linkage disequilibrium (LD), which involve association with T2D and MDD using the following models: dominant with complete penetrance (D1), dominant with incomplete penetrance (D2), recessive with complete penetrance (R1), and recessive with incomplete penetrance (R2). To test the presence or absence of LD blocks within the variants showing statistically significant results in T2D or MDD (*p* ≤ 0.05), we computed LD correlations via LD matrices among the SNPs available in the Toscani Italian population from the 1000 Genomes Project (https://www.internationalgenome.org/data-portal/population/TSI (accessed on 28 May 2022)) (LDmatrix function-RDocumentation,). The SNPs that significantly correlated (r [2] ≥ 0.9) with other SNPs were considered within the same LD block and labeled based on that unique LD block (e.g., Set 01 and Set 02). All SNPs that were not correlated with any other SNPs were designated as “Independent”.

## 4. Conclusions

Our study expanded the phenotypic spectra of melanocortin receptor genes. This is the first study to report risk variants in *MC1R*, *MC2R*, and *MC3R* genes in T2D. *MC2R* and *MC5R* genes are replicated in MDD; however, these appear with one novel variant each. Within our dataset, only the *MC2R* gene appears to confer risks for both MDD and T2D, albeit with different risk variants. To further clarify the role of the melanocortin receptor genes in MDD-T2D, these findings should be replicated in other ethnicities to improve our understanding of the comorbidity of MDD and T2D.

## Figures and Tables

**Table 1:** Melanocortin Receptor Genes: Risk SNPs for MDD and T2D.

Disease	Gene	Model ^1^	SNP	Position	Ref	Alt	Risk Allele	Consequence	LD Block	Previously Reported in MDD or T2D
**MDD**	*MC2R*	R1	rs111734014	Chr18-13898060	C	G	C	Intronic	Independent	Novel
	*MC5R*	D1	rs2236700	Chr18-13826392	C	G	G	Missense (p.F209L)	NA	Novel
**T2D**	*MC1R*	D1, D2	rs1805007	Chr16-89919709	C	T	C	Missense (p.R151G)	Independent	Novel
		R1	rs201192930	Chr16-89919622	G	A	G	Missense (p.V122M)	NA	Novel
		R2	rs2228479	Chr16-89919532	G	A	A	Missense (p.V92M)	Independent	Novel
	*MC2R*	R1, R2	rs104894660	Chr18-13885110	G	A	G	Missense (p.R137W)	NA	Novel
	*MC3R*	D1	rs3746619	Chr20-56248749	C	A	C	5′-UTR	Set01	Novel
	*MC3R*	D1	rs3827103	Chr20-56248973	G	A	G	Missense (p.V44I)	Set01	Novel
	*MC4R*	R1, R2	-	Chr18-60372302	C	T	C	Nonsense (p.W16X)	NA	Novel

^1^ Models: D1: dominant, complete penetrance; D2: dominant, incomplete penetrance; R1: recessive, complete penetrance; R2: recessive, incomplete penetrance.

## Data Availability

The data presented in this study are available upon reasonable request. The data are not publicly available due to privacy restrictions and lack of specific patients’ consent.
